# Catching the evader: Can monoclonal antibodies interfere with *Staphylococcus aureus* immune escape?

**DOI:** 10.1080/21505594.2017.1320012

**Published:** 2017-05-02

**Authors:** Jean-Philippe Rasigade

**Affiliations:** Centre International de Recherche en Infectiologie, University of Lyon, France

**Keywords:** complement pathway, immune evasion, immunotherapy, passive immunization, SCIN

Approximately 20% of human beings harbor *Staphylococcus aureus (S. aureus)* in their nares or other body sites.^1^ Most *S. aureus* carriers never develop infection while others usually suffer from mild skin and soft tissue infections (SSTIs). Under certain circumstances, however, *S. aureus* can gain access to deeper tissues or the blood and cause life-threatening conditions.^2^ So far, our most effective response to this aggression has been to poison the bacterium with bactericidal agents. This approach has saved countless lives, but it has also led to a constant arms race between our ability to develop new drugs and the ability of *S. aureus* to evolve antimicrobial resistance.^3^ Not only do we have no guarantee that we will eventually win this race against natural selection, but we are also increasingly concerned with the collateral damages that antimicrobial drugs cause to the bacteria of our microbiote and the environment.^4,5^

We face, thus, the urgent need to develop more sustainable strategies against infection. To put the arms race to an end, a crucial approach is to favor highly specific therapies that interfere only with the cause of the disease while limiting consequences on uninvolved organisms. Although trivial at first glance, this specificity objective has an important evolutionary underpinning: pathogen evolution does not select for severe disease.^6^ Deep-seated infection, either by leading to the rapid death of the human host or by limiting her social activity, lowers the odds of colonizing new hosts for the *S. aureus* population. From this standpoint, severe staphylococcal infections that do not favor transmission, such as bacteremia, should be considered as evolutionary accidents caused by bacteria escaping their ecological niche, the human surfaces. Targeting bacteria only outside of their niche should, thus, lower the ecological pressure of therapy given that the bacterial invaders are often already engaged in an evolutionary dead-end: they will eventually be cleared by the immune system, or die with their host.

The ecological niche of *S. aureus* is distinct from the tissues whose invasion leads to severe disease. Hence, therapeutic specificity can be considered not only from a taxonomic standpoind (the antimicrobial spectrum) but also from an anatomic one, by restricting the therapeutic action to deep tissues and vital organs, while leaving mucosae unaffected. Several technical means can be contemplated to achieve this goal, including targeted drug delivery.^7^ Nonetheless, the most efficiently targeted antimicrobial strategy is arguably that of our own immune system. In physiologic conditions, immunity rapidly clears bacteria from tissues while allowing the survival of microbiote inhabitants, including *S. aureus*. A treatment meant to assist the immune system to provide an effective and balanced response to aggression—that is, pathogen eradication without excessive inflammation—should impose minimal selection pressure on transmissible, non-invading bacteria, thus fulfilling the sustainability objective.

Restoring a complete immune activity against *S. aureus* is difficult, however, due to the exceptional ability of *S. aureus* to evade both the innate and adaptive immune systems.^8^ The recognition of *S. aureus* surface antigens by antibodies is hampered by the staphylococcal protein A, reducing opsonization and phagocytosis. When phagocytosis succeeds, *S. aureus* still manages to survive within the phagosome,^9^ disrupts its membrane or subverts the autophagic pathway, eventually killing the phagocyte.^10,11^ The activation of the complement pathway is hampered by several virulence factors such as the staphylococcal complement inhibitor SCIN.^12^ Several secreted cytotoxins and leukotoxins activate and lyse immune cells before they even reach the bacteria.^13^ The versatility of these immune evasion strategies is currently considered a major candidate explanation of the repeated failures of anti-*S. aureus* vaccine strategies in clinical trials.^14^

Nonetheless, clinical observations suggest that adaptive immunity does contribute to controlling staphylococcal infections. For instance, patients who develop *S. aureus* bacteremia with their own colonizing strain are less likely to die than non-colonized patients,^15^ and patients with high antibody titers against several staphylococcal toxins are less likely to develop severe sepsis during *S. aureus* infection.^16^ With the hypothesis that adaptive immunity affords patients protection from severe staphylococcal infections comes the hope to determine the right combination of antigens (in active immunization strategies) or antibodies (in passive immunotherapy) that will mimic or reinforce an effective immunity with preventive or curative objectives. Given that most staphylococcal virulence factors are neither necessary nor sufficient to cause severe infection, however, blocking only one factor is unlikely to afford universal protection.^17^ Alternatively, we might consider these pathogenic functions in a cumulative fashion, where each of them contributes independently to the probability and severity of infection. A multi-targeted, or polyvalent, intervention might aim at blocking these functions one after another until the probability (severity) of infection becomes low enough to achieve preventive (curative) efficacy.

To follow this research direction toward polyvalent immunotherapy, a crucial task is to rank the potential targets by their contribution to the condition we want to prevent. Many staphylococcal targets have been identified so far, including toxins, surface proteins or quorum sensing mediators. *S. aureus* toxins such as the α haemolysin (Hla) or the Panton-Valentine leukocidin (PVL), which are involved in lethal necrotizing pneumonia, have been understandably considered major candidates for both passive and active immunization strategies.^18^ However, concerns have been raised by several authors that immunization against PVL might be ineffective at preventing SSTIs,^19^ based on the clinical observation that elevated PVL antibody titers did not prevent recurrence of PVL-associated SSTIs and the more worrisome conclusion of animal models that PVL immunization might enhance such infections.^20,21^ Strikingly, a recent study of toxin production in colonizing, SSTI and bacteremia *S. aureus* isolates demonstrated that bacteremia isolates were significantly less toxic than their colonizing and SSTI counterparts,^22^ thus suggesting that toxin production might indeed decrease the ability of *S. aureus* to reach or survive within the bloodstream.

These results can seem counterintuitive from the usual viewpoint that virulence leads to severe infections. They become coherent, however, if we lean toward an evolutionary perspective and consider that colonization and SSTIs, not bacteremia or lethal pneumonia, contribute to the transmission and the long-term survival of staphylococcal populations. In the context of colonization and superficial infections, pro-inflammatory toxins such as Hla and PVL might have evolved not only as bacterial defense mechanisms, but also as warning signals meant to alert immune cells of tissue invasion and to preserve the host from an uncontrolled, lethal infection. In contrast, releasing the same pro-inflammatory toxins in a vital organ such as the lung, which threatens the host's life without favoring *S. aureus* transmission, is unlikely to have provided any evolutionary benefit. Although speculative and difficult to test, this dual-faceted interpretation of toxin activity is further supported by animal models in which blocking the inflammatory response induced by the interaction of Hla with its ADAM10 eukaryotic receptor abolished mortality in experimental pneumonia but strongly increased the dermonecrosis area in experimental SSTI.^23^ Collectively, these observations and their proposed interpretation imply that toxins should be considered with caution when used as immunotherapeutic targets: the curative use of anti-toxin antibodies probably limits inflammatory-induced damages in deep-seated infections,^18^ however their usefulness for preventing the infection is still uncertain.^21^

Contrary to pro-inflammatory toxins, non-toxic immune evasion factors have not been involved so far in mechanisms beneficial to the host, lowering the risk that their inhibition might be detrimental. Preclinical models of immunization against protein A,^24,25^ which captures the constant fragment of immunoglobulins, or the coagulase,^26^ which coats the bacterium with a phagocytosis-inhibiting fibrin shield, have shown promising results. In this issue of *Virulence*, Hoekstra et al. describe properties of a neutralizing monoclonal antibody against a major immune evasion factor of *S. aureus*, the complement inhibitor SCIN.^27^ The research strategy of the authors stemmed from the clinical observation that patients with the epidermolysis bullosa chronic skin disease, associated with long-term *S. aureus* colonization, develop bacteremia less frequently than would have been expected given their impaired skin barrier. In these patients, high antibody titers against several *S. aureus* antigens were hypothesized to confer protection against bacterial invasion. Monoclonal antibodies against *S. aureus*, including the so-called 6D4 antibody targeting SCIN, were then identified by collecting and screening B-cells of these naturally immunized patients. The 6D4 antibody binds to amino acid residues that overlap with the previously identified active site of SCIN. 6D4 treatment restored both the deposition of C3b of *S. aureus* surface and the complement-induced lysis of rabbit erythrocytes in the presence of SCIN, hence demonstrating a functional neutralizing activity of the complement-inhibiting function.

In spite of this SCIN-neutralizing activity, Hoekstra et al. prudently suggest that the 6D4 antibody might not be useful for therapeutic purposes given that SCIN-negative isolates still cause disease. This argument is undebatable as long as the 6D4 antibody is to be used alone for therapy. In the framework of polyvalent immunotherapy, however, the 6D4 antibody probably deserves more attention due to its specificity, *in vitro* efficacy, well-determined interaction with the SCIN active site and, importantly, its primary isolation from patients suspected to exhibit anti-*S. aureus* protective immunity. Hence, further research should determine whether and to which extent SCIN inhibition contributes to protect against *S. aureus* infection *in vivo*. Whether active or passive polyvalent immunotherapy will finally succeed and provide us with efficient and sustainable solutions against *S. aureus* infections is uncertain. Major technical obstacles must be overcome, such as the limited ability of animal models to predict the clinical efficacy of anti-*S. aureus* immunization approaches.^14^ Research groups and industries who have independently developed virulence-inhibiting strategies should also be encouraged to collaborate and examine the therapeutic potential of combined strategies. This research task is difficult and risky, but the long-term potential of its sustainability objective is probably worth the effort. Meanwhile, each contribution to the arsenal of *S. aureus* immune evasion-inhibiting agents, including the 6D4 antibody, should be regarded as a significant step toward this important objective.
